# Case report: Primary giant osteosarcoma of the heart protruding into the pericardial space in a young woman: a case of multimodal imaging

**DOI:** 10.3389/fonc.2025.1538856

**Published:** 2025-08-19

**Authors:** Ling Zhao, Fei Li, Wenzhe Xu, Zhennian Zhao, Xiaoguang Huo, Yongting Zhang

**Affiliations:** ^1^ Zibo Central Hospital, Department of Ultrasound, Zibo, Shandong, China; ^2^ Zibo Hospital of Shandong Healthcare Group, No.1 Department of Cardiology, Zibo, Shandong, China; ^3^ Zibo Hospital of Traditional Chinese Medicine, Special Inspection Department, Zibo, Shandong, China

**Keywords:** primary cardiac osteosarcoma, echocardiography, MRI enhanced scan, PET-CT, pathology

## Abstract

**Background:**

Primary cardiac malignancies are rare, and primary cardiac osteosarcoma is even rarer. The prognosis is poor. Four multimodality imaging data of the same case have not been retrieved yet.

**Case:**

We report a 21-year-old woman with osteosarcoma in the left atrium, atrial wall, and pericardial cavity. She was hospitalized for cholecystitis and chest tightness. Transthoracic echocardiogram (mixed echogenic tumor in the left atrium and pericardium), computed tomography (CT), cardiac magnetic resonance imaging (MRI) enhancement scan (heterogeneous enhancement of tumor), and positron emission tomography (PET)-CT examination (high FDG uptake of tumor) revealed mass in the left atrium, atrial wall, and pericardial cavity. According to the comprehensive analysis of multimodality imaging examination results, it was considered as a cardiac malignant tumor. A surgical plan was immediately formulated and surgical treatment was performed. The patient developed renal failure after operation. After active hemofiltration, anti-infection, anticoagulation, expectorant, cardiotonic, diuretic, nutritional support, and other symptomatic treatments, her condition was improved, and she was discharged after continuous treatment. After nine cycles of chemotherapy, the patient has been followed up for 11 months. The patient is in good condition and no recurrence has been observed.

**Conclusion:**

Primary cardiac osteosarcoma is extremely rare. Multimodality imaging, including echocardiography, CT, MRI, and PET-CT, has a high diagnostic value for cardiac osteosarcoma, which can provide reference for the feasibility and safety of surgical operation.

## 1 Introduction

Primary cardiac sarcomas are extremely rare cardiac malignancies. Angiosarcoma is the most common histologic type, followed by undifferentiated high-grade pleomorphic cardiac sarcomas. Osteosarcoma and chondrosarcoma are extremely rare types. Primary cardiac osteosarcoma generally has a poor prognosis due to tumor invasion, including surgery and medical treatment. To date, there is a lack of standardized guidelines for the diagnosis and treatment of primary cardiac osteosarcoma, and multimodal imaging data of the same patient are extremely rare.

## 2 Case report

**Figure 1:**
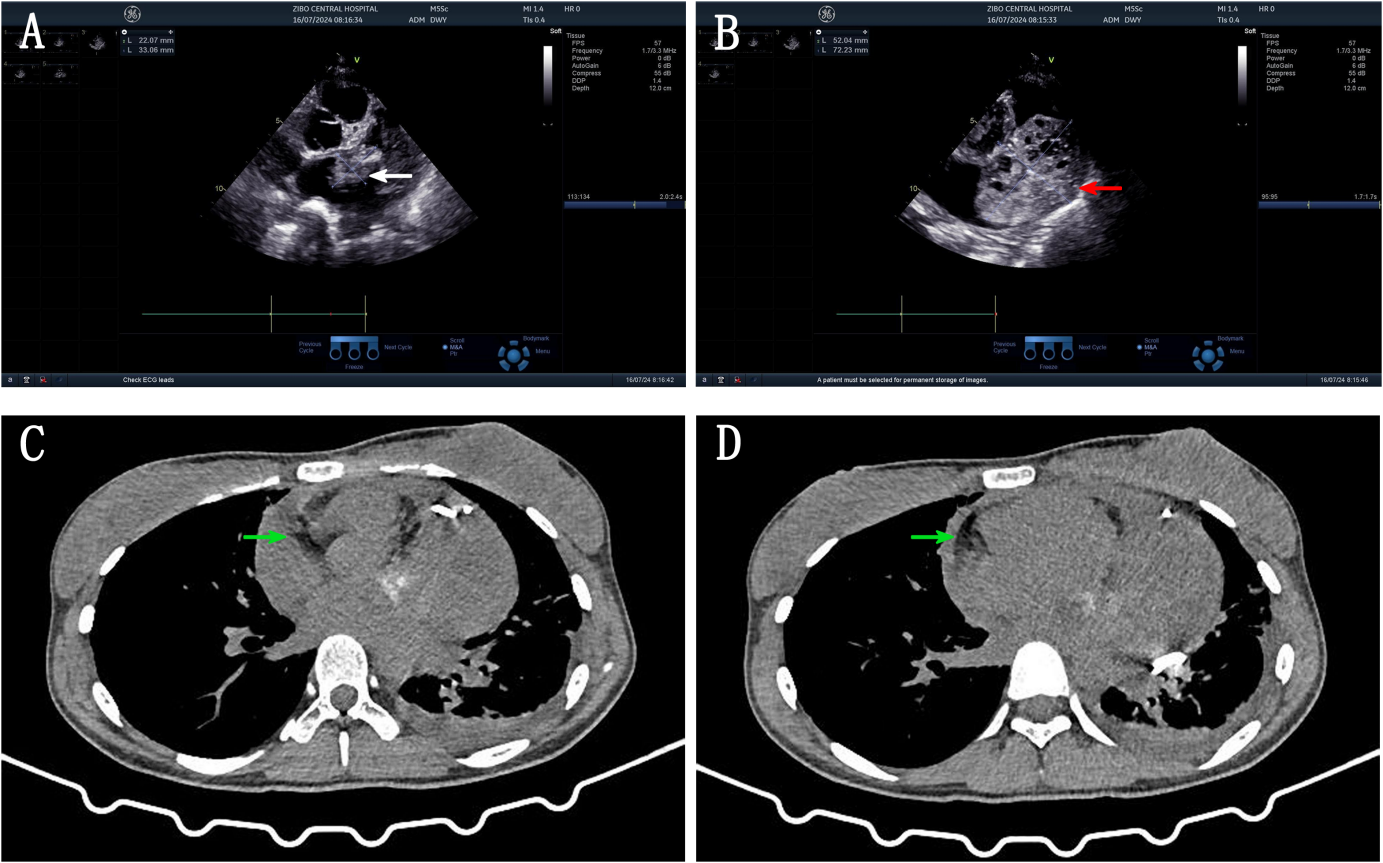
**(A)** US image: cystic solid mixed echo mass in the left atrium attached to the lateral wall of the left atrium (white arrow). **(B)** US image: cystic solid mixed echogenic mass in the lateral pericardial cavity of the left ventricular sidewall, connected with a left atrial mass (red arrow). **(C, D)** CT image: High and low mixed density shadows were seen in the left atrium and left pericardium, irregular shape, unclear boundary with pericardium, calcification was seen locally, consolidation shadow was seen in left lower lung, and a small amount of effusion was seen in left pleural cavity (white arrow).

**Figure 2:**
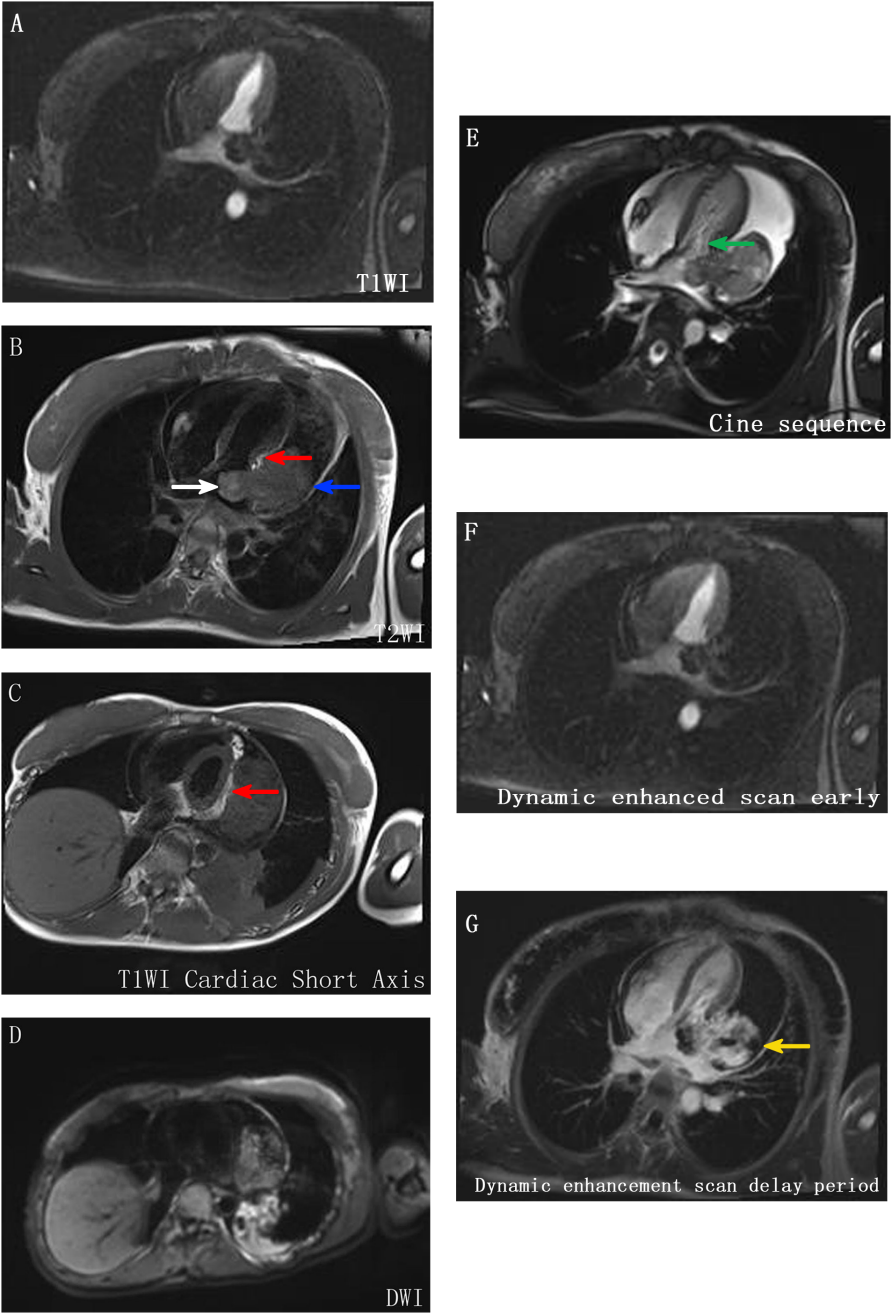
MR image. **(A–E)** A mass was seen in the lateral wall area of the left atrium and its periphery, with unclear boundary and irregular shape, showing slightly longer T1 and T2 signal shadow, longer T2 signal in the interior, short T2 signal in the edge (indicating hemorrhage), uneven high signal in T2WI lipid pressure, high signal in DWI, poor activity of lesion, main body located outside the left atrium (blue arrow), part protruding into the left atrium, resulting in effective volume reduction (white arrow), and anterior involvement of the mitral valve orifice area (green arrow). The left ventricle is also compressed (indicated by red scissors), and the volume of the adjacent left ventricular chamber is slightly reduced. **(F, G)** The perfusion enhancement is not obvious in the first pass of enhanced scan, and the heterogeneous enhancement changes are obvious in delayed enhancement (indicated by the yellow arrow).

**Figure 3:**
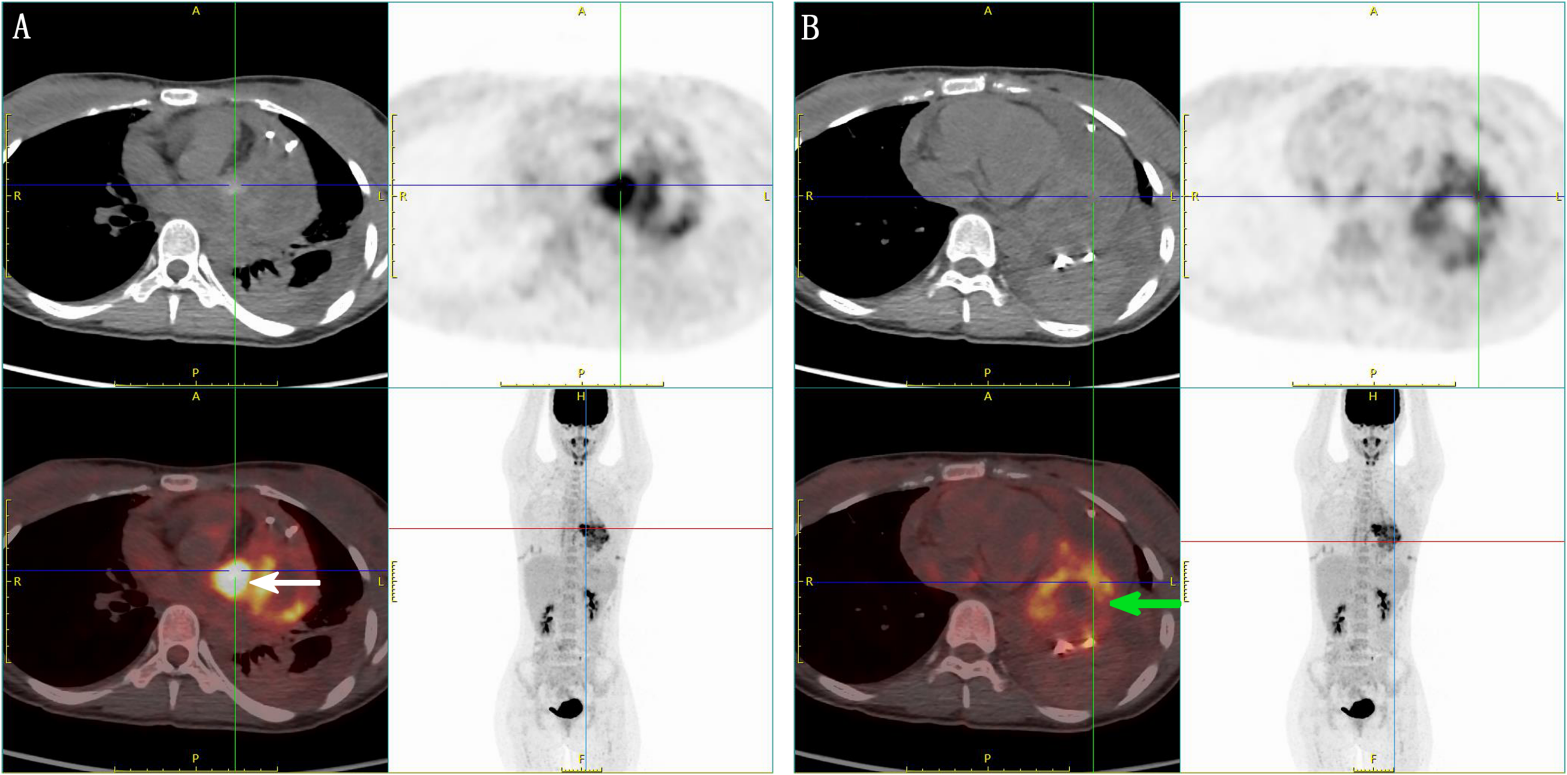
PET-CT image. **(A)** Enlarged cardiac shadow, a mixed high-density mass with obviously increased FDG metabolism protruding into the pericardium on the lateral wall of the left atrium, showing an irregular shape, the high-metabolism part mainly located in the left atrium SUVmax = 7.6 (indicated by the white arrow), thickened pericardium, with more liquid density shadow inside, and no obvious FDG uptake. **(B)** The intrapericardial fraction SUVmax = 5.0 (indicated by the green arrow) has a lower FDG metabolism than the left atrial fraction.

A 21-year-old female patient was admitted to the hospital on 10 July 2024. The chief complaint: The patient felt chest tightness and discomfort when receiving infusion treatment in a local hospital due to acute cholecystitis. Cardiac color Doppler ultrasound showed that there was a mass in the left atrium, which was considered to be thrombosis, so she came to our hospital urgently. The patient was usually in good health and had no medical history related to the disease. Physical examination: The thorax was symmetrical, the respiratory movement was symmetrical on both sides, the sound on percussion was unvoiced, the breath sounds in both lungs were low, and no dry or wet rales were heard. There was no abnormal protrusion in the precordial area, the apex beat was unclear, and no tremor or lifting pockets were found. There was no significant expansion of the relative dullness boundary of the heart on percussion. Heart rate was 90 beats/min, with a regular rhythm, distant heart sounds, and no pathological murmur in precordial area, and P2 was not hyperactive. Femoral artery had a gunshot sound (1), with no pestle toe. Transthoracic ultrasound examination: A cystic and solid mixed echo mass was detected in the left atrium, with a range of approximately 3.3 × 2.2 cm, attached to the lateral wall of the left atrium, without obvious mobility; a cystic and solid mixed echo mass was detected in the lateral pericardial cavity of the lateral wall of the left ventricle, with a size of approximately 7.2 × 5.2 cm, seemingly connected to the mass in the left atrium, without obvious mobility. A large amount of fluid dark areas were detected in the pericardial cavity. The depth of left ventricular posterior wall effusion was 2.4 cm, and the depth of left ventricular lateral wall effusion was 2.8 cm. A mixed echo mass in the left atrium, a mixed echo mass in the pericardial cavity (considered as cardiac tumor), and pericardial effusion (large amount) were suggested (**Figures 1A, B**). Computed tomography (CT) examination: High and low mixed density shadow (CT value approximately 33 Hu) can be seen in the left atrium and left side of the pericardium; the size is approximately 7.8 × 5.3 cm, with an irregular shape and unclear boundary with pericardium; local calcification can be seen, consolidation shadow can be seen in the left lower lung, and a small amount of effusion can be seen in left pleural cavity. Prompt: Left atrium and pericardium left side mixed density focus, considered cardiac mass combined with pericardial hemorrhage, local unclear boundary with pericardium, left lung compression, and left pleural effusion (**Figures 1C, D**). Magnetic resonance imaging (MRI) examination: A mass was seen in the lateral wall area of the left atrium and its periphery. The larger section size was approximately 7 × 4.7 × 6.2 cm. The boundary was unclear and irregular. It showed a slightly longer T1 and a slightly longer T2 signal shadow. A longer T2 signal was seen in the interior. A short T2 signal was seen at the edge (indicating hemorrhage). T2WI fat pressure showed uneven high signal. DWI (diffusion-weighted imaging) showed a high signal. The lesion activity was poor. The main body was located outside the left atrium. Some of them protruded into the left atrium, resulting in reduced effective volume. The mitral valve orifice area was involved forward. Left ventricular compression was also seen, the volume of adjacent left ventricular chamber decreased slightly, perfusion enhancement was not obvious in the first pass of enhanced scanning, and heterogeneous enhancement was obvious in delayed enhancement. Pericardial effusion was seen, and a small amount of pleural effusion was seen on the left side. The initial T1 value of the lesion increased, but the T1 value of enhanced scan decreased significantly. Strip-shaped and round short T1 and short T2 signal shadows were also seen in the lateral wall of the right atrium and right ventricle, and the enhancement was not obvious on enhanced scan. It is suggested that the space-occupying lesions in the lateral wall of the left atrium and its surroundings are consistent with tumor manifestations and should be considered sarcomas or pheochromocytomas. Pericardial effusion and small pleural effusion were present on the left side. There were multiple localized abnormal signals in the lateral wall of the right atrium and right ventricle, which were considered hemorrhage (**Figure 2**). Positron emission tomography (PET)-CT examination: The cardiac shadow was enlarged, and a mixed high-density mass with obviously increased FDG metabolism was seen protruding into the pericardium on the lateral wall of the left atrium, showing an irregular shape, with an area of approximately 7.0 × 4.8 × 7.6 cm. The hypermetabolic part was mainly located in the left atrium, SUVmax = 7.6 (in the left atrium) and SUVmax = 5.0 (in the pericardium): no abnormal FDG metabolism increased obviously abnormal FDG metabolism increased focus was found in the remaining cardiac cavities; pericardium thickened, with more liquid density shadows and FDG uptake was not evident. It is suggested that the lateral wall of the left atrium is mixed with a high-density mass, occupying the atrium and protruding into the pericardium; FDG metabolism is unevenly increased, and combined with medical history, it is considered as a primary malignant disease; pericardial effusion (**Figure 3**).

**Figure 4:**
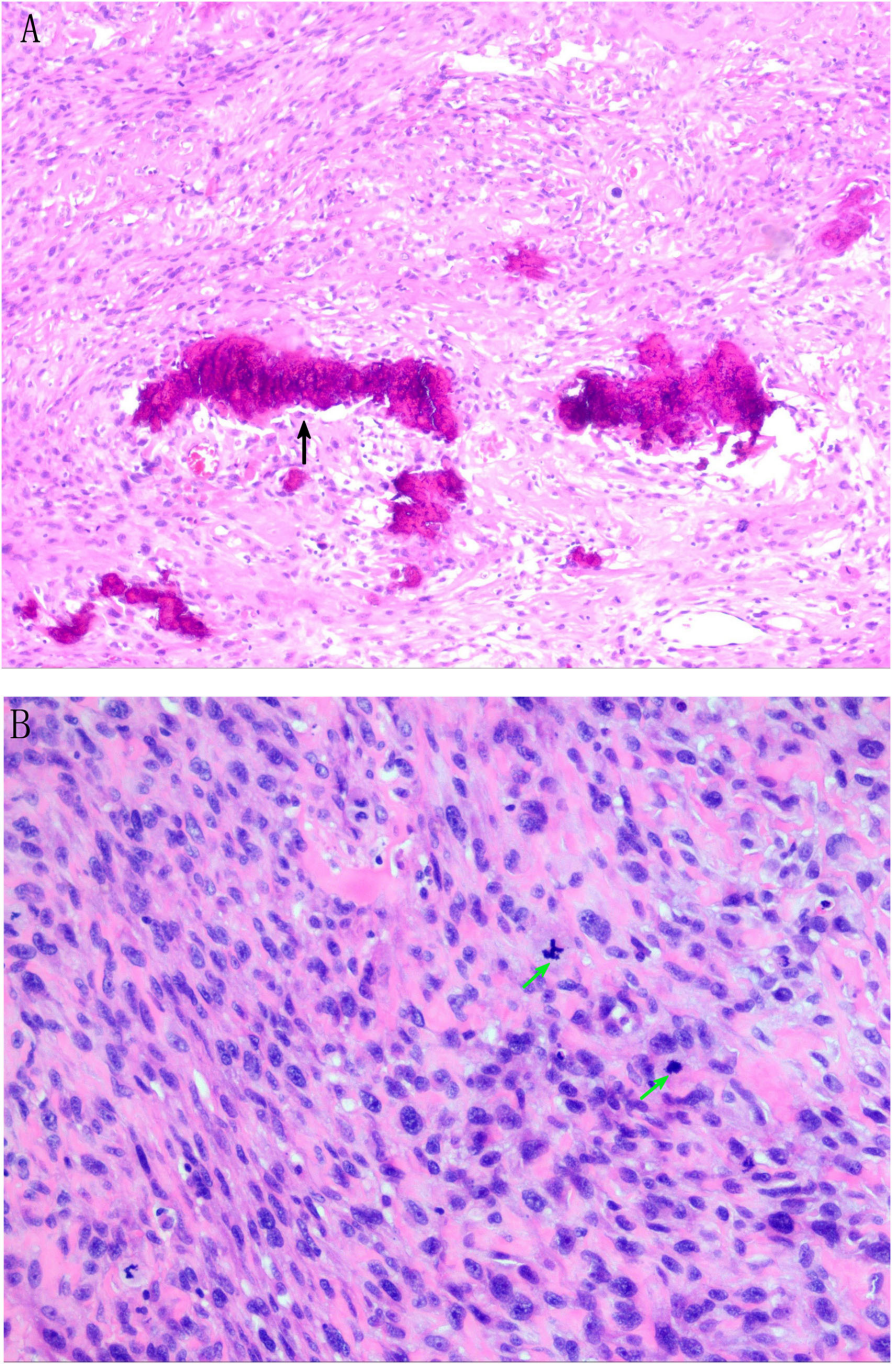
Pathological result description. **(A)** Tumor cells are composed of proliferating epithelioid and short spindle cells with increased nuclear-to-cytoplasmic ratio and mitotic figures (black arrows). **(B)** Tumor cell nuclei are dark stained, round, or oval, with obvious heteromorphism, and pink stained osteoid matrix can be seen in the stroma (green arrow). Meaning of each indicator in immunohistochemical results: CK(−): Non-epithelial tumor Vimentin(+): Support mesenchymal tissue-derived tumors P53(wild type): The P53 gene is not mutated and the prognosis is relatively good KI67 (hot spot area approximately 40+): High proliferation index, indicating active tumor cell division and high malignant degree SATB2(+): Specific marker for osteoblast differentiation S100 (a few cells weakly positive): A few cells weakly positive, excluding typical neurogenic tumors CD34(−), ERG(−): Exclude vascular tumors CD99(+): Common in small round cell tumors, auxiliary diagnosis but needs to be combined with other indicators SMA (focal +): Focal positive for smooth muscle actin, suggesting a small amount of myogenic differentiation.

On 13 July 2024, ultrasound-guided pericardial effusion catheter drainage was performed, and the drainage fluid was a dark red bloody non-coagulable fluid, with a high risk of continued bleeding of tumor. Laboratory test results: Pericardial effusion cancer cells: none; CRP: 70.8800 mg/L (0.00–5.00); CA-125: 62.9000 U/mL (0.00–35.00); N-terminal B-type natriuretic peptide precursor: 195.0000 pg/mL (0–125); D-dimer (DDU): 1.1500 mg/L (0.00–0.50). Preoperative discussion: The tumor is large and considered malignant, which may not be suitable for thoracoscopic minimally invasive therapy and interventional therapy. The patient has developed chest distress symptoms, and it is not advisable to wait for observation. It is considered that traditional thoracotomy is the best choice, and radical resection of the tumor to the maximum extent during surgery is the key. Considering that the tumor has invaded the left atrial wall, mitral valve, and pericardial cavity, reconstruction of the left atrium, mitral valve replacement, and pericardial fenestration should be performed after tumor resection. Further chemotherapy and radiotherapy were determined according to the pathological results. Subsequently, after excluding distant metastasis and contraindications and adequate and comprehensive preoperative preparation, cardiac tumor resection + left atrial reconstruction + mitral valve replacement + pericardial fenestration was performed under general anesthesia, hypothermia, and cardiopulmonary bypass on 28 July 2024. Pathological examination confirmed osteosarcoma in the left atrium, atrial wall, and pericardial cavity. Immunohistochemical results: CK(−), vimentin(+), P53 (wild type), KI67 (hot spot region ~ 40+), SATB2(+), CD99(+), S100 (a few cells weakly +), SMA (focal +), CD34(−), and ERG(−) (**Figure 4**). The patient was complicated with acute renal failure after operation and was given bedside hemofiltration, anti-infection, anticoagulation, expectorant, cardiotonic, diuretic, nutritional support, and other treatments. The patient’s condition improved. After returning to the ward, the patient was complicated with fever, throat discomfort, and nasal discharge. Molecular test report on 22 August 2024: novel coronavirus (SARS-CoV-2) nucleic acid screening ORF1ab group positive ↑. Symptomatic treatment was given, and the patient recovered well after operation. Reexamination showed creatinine 73.0 μmol/L and urea nitrogen 5.5 mmol/L. After continuous treatment, the patient had normal temperature, no fever, no cough and expectoration, and no chest distress and suffocation, and was discharged from the hospital. Combined with postoperative pathological results, the patient was given alternating chemotherapy with an HDMTX (high-dose methotrexate)/HDIFO (high-dose ifosfamide)/AP (liposomal doxorubicin + cisplatin) regimen. At present, three rounds of chemotherapy with a total of nine cycles have been performed, and the patient is in good condition at present.

## 3 Discussion

Cardiac tumors are classified into primary and secondary (metastatic) tumors. Primary cardiac tumors are extremely rare clinically, and a few are malignant (1). Metastasis is the most common cardiac malignancy (2). Primary cardiac malignancies are rare and lack specificity in clinical manifestations. They are mostly found in autopsy reports, with an incidence of approximately 0.001% to 0.280% (3). Osteosarcoma accounts for approximately 10% of primary cardiac malignancies (4, 5). Primary cardiac sarcomas are prone to metastasis, with the lungs being the most common site, and can also metastasize to soft tissues (including the mediastinum), bones, brain, liver, thyroid, intestine, peritoneum, and skin (6, 7). Primary cardiac osteosarcoma is slightly more common in women than in men, with an average age of 50 to 60 years at diagnosis (8). This case, a 21-year-old woman, is even rarer.

Primary cardiac osteosarcoma and benign tumors mostly occur in the left atrium, while metastases from osteosarcoma at other sites to the heart are most commonly located on the right side of the heart (5, 11). There is no evidence that primary cardiac osteosarcoma is malignant from benign tumors. The cell of origin of the tumor has not been clearly identified. At present, most researchers believe that they originate from undifferentiated mesenchymal stem cells in the intima, which transform into viable osteoblasts secondary to overexpression of specific genes (8–10).

The final diagnosis of cardiac osteosarcoma requires histology and immunohistochemistry. Osteosarcoma is a heterogeneous group of tumors containing malignant osteogenic cells (8). Macroscopically, primary cardiac osteosarcoma may appear soft and granular (osteolytic) or osteoporotic and dense (osteoporosis), depending on the degree of mineralization (11). Calcification, necrosis, or hemorrhage could be seen within the tumor. Histologically, the tumor contains varying numbers of spindle cells, osteoid, bone, and cartilage. It rarely occurs in soft tissues (7, 12). Malignant and benign osteosarcoma can be divided into osteoblast type, chondroblastic type, and fibroblast type according to its main components. Several previous studies have established similarities between the immunophenotypes of extraosseous and skeletal osteosarcoma. These tumors were uniformly positive for vimentin and some were positive for α-actin smooth muscle. Histological grading has been reported to predict a patient’s final prognosis, and increased nuclear apoptosis often predicts a poor prognosis (8).

Benign cardiac tumors have a good prognosis after surgery, but malignant cardiac tumors have a poor prognosis. Therefore, early noninvasive examination is very important to distinguish the nature of cardiac tumors. Imaging examination is a commonly used auxiliary examination method for cardiac tumors (13). Echocardiography is easy to operate and is often used as the first screening method for cardiac tumor examination. Enhanced CT can obtain various morphological structure characteristics and blood supply characteristics of cardiac tumors. MRI (14) has excellent soft tissue resolution and blood flow imaging capabilities, which can well evaluate the extent of tumor invasion of myocardium, usually showing isointensity on T1-weighted imaging and heterogeneous or high intensity on T2-weighted imaging. The MRI delayed enhancement features of osteosarcoma were significantly correlated with histopathological features, and the core mechanism was derived from abnormal neovascularization and extracellular matrix remodeling. The enhancement degree of delayed phase was positively correlated with microvessel density (MVD) in histopathology. Neuropilin-1 (NRP-1) is highly expressed in osteosarcoma vascular endothelial cells. This receptor leads to structural destruction of vascular basement membrane by enhancing the activity of the vascular endothelial growth factor (VEGF) signaling pathway. Increased vascular permeability is the main driver of delayed reinforcement. Radionuclide scans of the whole body can identify the presence of neoplastic lesions elsewhere. ^18^F-FDG PET-CT imaging has been widely used in the diagnosis and monitoring of malignant tumors on the basis of anatomical images and metabolic information of tumor cells. Malignant cardiac tumors usually grow faster and have a high metabolism, which is characterized by the high uptake of FDG. Therefore, they are widely used in the diagnosis of lung cancer, lymphoma, breast cancer, and other malignant tumors. However, because of the rarity of cardiac tumors, relevant clinical studies are rare and mostly limited to studies on FDG uptake by tumors (15, 16). For example, Rahbar et al. (15) once proposed SUVmax = 3.5 as the threshold for differential diagnosis of benign and malignant diseases, and the diagnostic sensitivity can reach 100% and the specificity can reach 86%. Osteosarcoma is a highly malignant bone tumor, and its PET-CT normalized uptake value (SUVmax) is positively correlated with tumor metabolic activity. In osteosarcoma, SUVmax ≥ 8.0 usually indicates that the tumor is in a hypermetabolic state, corresponding to a pathological KI67 index > 30% (*p* = 0.01), which can be used as a quantitative threshold to distinguish poorly differentiated from well-differentiated subtypes.

At present, most literature reports on cardiac malignant tumors only have one to two examinations. Considering that the patient is relatively young, comprehensive multimodal imaging has been performed in our case. The patient has been examined comprehensively by CT, echocardiography, enhanced MRI, and PET-CT successively. Echocardiography can observe not only the size, location, and relationship between the tumor and heart wall and valve, but also the influence of tumor on the inflow and outflow tract, and detect whether there is hemodynamic change caused by the tumor, which provides a basis for clinical diagnosis, preoperative evaluation, and selection of operation mode. CT examination was performed in this patient before echocardiography examination, and CT showed a left atrial and pericardial mass with intrapericardial hemorrhage. Then, echocardiography examination was performed, and it was found that the tumor in the left atrium was closely connected to the lateral wall of the left atrium and pericardial mass with a large connection area and accompanied by a large amount of pericardial effusion, which was considered as invasive growth possibility of a malignant tumor. Pericardiocentesis was performed under ultrasound guidance, and the drainage fluid was dark red and bloody. Contrast-enhanced MRI showed that the first pass perfusion enhancement was not obvious, and the delayed perfusion enhancement was obvious and heterogeneous. Areas of heterogeneous enhancement suggest areas of cellular proliferation (7). PET-CT scans allow diagnostic examination of primary cardiac malignancies and exclude the absence of other active neoplastic disease for improved staging and surveillance (12). PET-CT scan was performed in this patient, suggesting the primary site of the tumor protruding into the pericardial cavity. SUVmax = 7.6 in the left atrium, close to 8.0, KI67 (hot spot area approximately 40+), fully indicating that the tumor belongs to a high metabolic state. The comprehensive examination of this case confirmed that multimodality imaging combined with examination can help diagnose cardiac space-occupying lesions, guide clinical early surgical resection and other treatment options, and improve the prognosis of patients.

Owing to the rarity of primary cardiac malignancies, there are currently no accepted treatment guidelines. Surgical treatment can reduce intracardiac obstruction, and adjuvant chemotherapy after surgical resection can reduce patient mortality compared with surgery alone (17). Cardiac malignancies have a poor prognosis. We provide adequate humanistic care to patients and their families to help them choose more appropriate treatment options according to existing medical treatment methods. First of all, it is suggested that surgery should be performed within a limited time, adjuvant chemotherapy and radiotherapy should be performed after surgery, and alternative treatment options should be explained. Alternative 1: Drug treatment can only temporarily relieve symptoms and cannot remove tumor lesions. Alternative 2: Watch and wait. There is a risk of disease progression, distant metastasis, and missing the best time for surgery. Alternative 3: Heart transplantation, but there is a long waiting time for organs and there are practical implementation difficulties. The patient eventually chose surgery and postoperative chemoradiation. According to the results of multimodal imaging examination, the tumor was staged after excluding metastasis from other sites. After comprehensive evaluation, surgical treatment was performed. The postoperative pathological results were osteosarcoma in the left atrium, atrial wall, and pericardial cavity. In order to further confirm the diagnosis and follow-up treatment, gene detection was suggested for the patient, but the patient’s family refused.

Primary cardiac osteosarcoma is a rare clinical disease with few case reports. It is most common in the left atrium. It is aggressive and prone to metastasis. Recurrence and metastasis are common features of cardiac osteosarcoma with poor prognosis. FISH analysis of a rare case of primary cardiac fibroblastic osteosarcoma revealed MDM2 gene amplification (8, 18). Patients with osteosarcoma are usually unable to undergo complete surgical resection due to invasion of important structures, and it has been suggested that even if surgical resection is successful, the prognosis of osteosarcoma patients with or without chemotherapy after surgery is still poor (19, 20). The mean survival of reported cases ranged from 7 weeks to 72 months. The main cause of death is local and/or systemic disease recurrence (5, 21). Postoperative chemotherapy and/or radiotherapy, if metastatic, can improve prognosis in patients with metastatic disease and in patients with local recurrence (6). However, the incidence of cancer is low, the number of cases is small, and there is currently no standard treatment, and because the myocardium is less resistant to chemotherapy, surgery remains the treatment of choice for cardiac osteosarcoma and is the only method currently proven to prolong survival in such patients. Recently, heart transplantation can be used in patients with unresectable tumors without distant metastases (8).

## 4 Conclusion

Primary cardiac osteosarcoma is a rare malignant tumor with poor prognosis. Currently, there are no universally accepted guidelines for its diagnosis and treatment. We performed multimodality imaging examination to improve the imaging understanding of this disease, explore the application experience of multimodality imaging technique in the auxiliary diagnosis of cardiac osteosarcoma, provide reference for the operability and safety of surgery, and provide reference materials for further clinical research of primary cardiac osteosarcoma. The patient has been followed up for 11 months. On 11 September 2024, reexamination showed multiple small lymph nodes in the mediastinum, bilateral axilla, and supraclavicular region; the larger one was approximately 0.7 × 0.9 cm. There was no significant change in the three subsequent reexaminations on 12 November 2024, 23 January 2025, and 14 April 2025. No obvious recurrence signs were found in other parts. At present, the patient is in good condition and may have benefited from a comprehensive preoperative imaging examination, the rapid formulation of a surgical plan, no distant metastasis of tumor, and complete surgical resection.

## Data Availability

The original contributions presented in the study are included in the article/supplementary material. Further inquiries can be directed to the corresponding author.
